# Operations on the Antrum

**Published:** 1851-01

**Authors:** R. D. Addington


### ARTICLE IX.

*Operations on the Antrum.* By R. D. Addington, D. D. S.

Early in March, 1850, whilst I was endeavoring to prevail on a gentleman of this city, to submit to the extraction of some diseased teeth, as the first step to be taken to restore his mouth to health, I casually mentioned some of the symptoms which occur from a diseased antrum; when he informed me, he had an intimate friend, who, he had no doubt, from my description, was suffering from it. He exhibited much pleasure, when I assured him the disease might be readily cured, and he promised to persuade his friend to see me. In this, he anticipated much difficulty, as his friend had patiently submitted to his disease for about twelve months, and was unwilling to make his case known; yet felt convinced the disease was undermining his health. His relations had often exhibited much concern about him, as he had a large family dependent on him for a support.

With this information, I was the better prepared to receive a visit from this gentleman, who had heretofore shunned advice from a false delicacy. Some days after, I had the pleasure of meeting Mr. S. at my office. He was about forty-five years of age and apparently in bad health. He informed me that an offensive and nauseating discharge continually dripped from his right nostril. This, I found, had obviously assumed an unusual malignancy, which I attributed to the enervated condition of his body. He informed me, the discharge was sufficient to thoroughly dampen several handkerchiefs a day. When his attention was first drawn to the disease, he felt an uneasiness about the right cheek. This was soon followed by violent pains, which sometimes continued, at other times, soon passed off; and when he began to notice the discharge from his nostril he was only occasionally troubled with a dull pain, accompanied with fullness. This cessation of excruciating pain had reconciled him to the excessive discharge, which, from its acrid character, had caused the pituitous membrane of the nose to be much inflamed. An examination of the mouth and teeth presented an unusual degree of health, except the second and third molars of the right side, the crowns of which had been destroyed by caries, and their roots separated and showing five pieces; the gum around them was inflamed, it being more decidedly flabby about the roots of the second molar. When the head was inclined to the left, the discharge from his nostril was increased. There were no indications to suspect a scrofulous or scorbutic disposition or those of a venereal taint. I viewed the case as a chronic disease of the antrum, the cause entirely local, and determined to treat it accordingly. I, therefore, assured my patient of a speedy cure, if he would allow me to remove the roots. To which he assented. After their extraction, I found no connection through the alveoli with the antrum, except by the palatal alveolus of the second molar. The root from this cavity was of ordinary length, without the appearance of absorption, and could not have perforated the antrum. The intervening plate of bone that once existed, as with the other roots, had been destroyed by a puriform secretion, generated in an abscess, I found attached to the root. This connection I enlarged, which was followed by a small discharge. I injected the antrum through it with cold water, when I observed passing into the pharynx a large quantity of matter, mixed with flocculi. The odor was now insufferable to the patient and myself; we, therefore, retreated from the apartment, and he was requested to call the next day—when the same result followed the injection of cold water, as on the preceding day. On the third, the discharge was less offensive. The patient was informed of the importance of preventing the closure of the opening; and as I considered this the best remedy for a cure, I desired him to enter his syringe three times a day, and inject the cavity with the following formula, to remove the fetor of its secretions:

℞ ℥½ chloride of soda.
℥2 rose water.

